# DSPose: Dual-Space-Driven Keypoint Topology Modeling for Human Pose Estimation

**DOI:** 10.3390/s23177626

**Published:** 2023-09-03

**Authors:** Anran Zhao, Jingli Li, Hongtao Zeng, Hongren Cheng, Liangshan Dong

**Affiliations:** 1 School of Remote Sensing and Information Engineering, Wuhan University, Wuhan 430079, China; anranzhao415@whu.edu.cn; 2 School of Physical Education, Huazhong University of Science and Technology, Wuhan 430074, China; zenghongtao@hust.edu.cn; 3 Sport and Health Initiative, Optical Valley Laboratory, Wuhan 430074, China; 4 Sports Big-Data Research Center, Wuhan Sports University, Wuhan 430079, China; hongrencheng0718@163.com; 5 School of Physical Education, China University of Geosciences, Wuhan 430074, China; dlsty2014@126.com

**Keywords:** human pose estimation, Transformer, graph convolutional network, dual space, keypoint detection

## Abstract

Human pose estimation is the basis of many downstream tasks, such as motor intervention, behavior understanding, and human–computer interaction. The existing human pose estimation methods rely too much on the similarity of keypoints at the image feature level, which is vulnerable to three problems: object occlusion, keypoints ghost, and neighbor pose interference. We propose a dual-space-driven topology model for the human pose estimation task. Firstly, the model extracts relatively accurate keypoints features through a Transformer-based feature extraction method. Then, the correlation of keypoints in the physical space is introduced to alleviate the error localization problem caused by excessive dependence on the feature-level representation of the model. Finally, through the graph convolutional neural network, the spatial correlation of keypoints and the feature correlation are effectively fused to obtain more accurate human pose estimation results. The experimental results on real datasets also further verify the effectiveness of our proposed model.

## 1. Introduction

Human pose estimation is always a challenging problem in machine vision. Its main goal is to determine the spatial position of a person’s body keypoints from a given image or video. It is also the basis for many high-level semantic tasks and downstream application scenarios, such as motion intervention, human–computer interaction [1,2,3], intelligent education, museum immersive experiences, etc.

Considering the overall framework of the algorithm, 2D multi-person human pose estimation can be divided into two categories: bottom–up and top–down estimation methods. Bottom–up estimation methods take the original image as input, first estimate all the keypoints coordinates in the figure, and then divide the keypoints coordinates according to the human body, so as to generate 2D human pose estimation corresponding to each body. The top–down method first detects the human body and then predicts the keypoints coordinates for each detected human body. The original problem of multi-person human pose estimation is transformed into multiple single-person human pose estimation tasks, simplifying the complexity of keypoints estimation and improving the accuracy of human pose estimation to a certain extent. Therefore, it is favored by the majority of researchers. The method proposed in this paper also belongs to the top–down approach.

In addition, human pose estimation methods are divided into CNN-based and Transformer-based approaches. Generally speaking, the CNN-based methods [4,5,6] are mainly divided into two stages: feature extraction and keypoints regression. Among them, the feature extraction part mainly extracts the image features through different backbones, to extract the human body information in the images. The keypoints regression part maps the features in the images to the coordinates of the keypoints through the regression model and then deduces the pose. In this stage, a deconvolution layer is added to the output layer to generate heatmaps for keypoints prediction, which can further improve the model’s performance. Since Transformer are widely used in natural language processing and computer vision, Transformer-based methods [7,8,9,10] are also popular in human pose estimation currently. These methods often combine conventional feature extraction networks with Transformer models. A multi-layer attention-sensing module is used to capture the feature connections between the keypoints of the images, and better results are achieved with fewer parameters.

Although these top–down pose estimation methods have achieved good results, the accurate identification of keypoints of the human body still faces three challenges: object occlusion (object occlusion leads to the loss of information of part of the keypoints), keypoints ghost (portrait ghost in the shooting process), and neighbor pose interference (neighbor keypoints interference). The three challenges mainly come from the fact that the model relies too much on the correlation of keypoints in feature space and ignores the correlation of keypoints in physical space, which may have a negative impact on keypoints detection. In physical space, the keypoints of the human body follow certain distribution laws, so this knowledge is also crucial for posture modeling.

In this paper, the above correlations are divided into the following two categories: (1) the correlation of keypoints in physical space refers to the overall distribution relationship followed by keypoints in the human body, which is based on people’s common sense of life and the comprehensive statistics of datasets such as the rigid body characteristics of the keypoints of the head and the symmetry of the limbs; (2) the correlation of keypoints in feature space refers to the pixel-level feature relationship between keypoints of the human body in a single image. However, the keypoints of the human body in a single image may be different from the physical space due to factors such as occlusion, distortion caused by shooting methods and neighbor interference. To visualize these two distinct relationships, we used different ways to model the two relationships separately. For the correlation of keypoints in physical space, we use the results based on human physiological structure and statistical analysis to construct the topological structure of physical space. For the relevance of keypoints in feature space, we first use the Transformer-based network to extract keypoints features. Then, we calculate the similarity degree of extracted keypoints features to represent the relevance of feature space. Finally, the image convolutional neural network effectively integrates the spatial correlation and the feature correlation to obtain more accurate human pose estimation results.

Overall, our method makes the following contributions:

1. We proposed a novel Dual-Space Driven Keypoint Topology Modeling for human pose estimation. The correlations of physical space and feature space are introduced into the model to improve the accuracy of the model further.
2. We extract the feature relations based on the keypoints feature extracted by Transformer. We also combine human physiological structure and statistical analysis to express the correlations of keypoints of human bodies in real world and figures. We finally use graph convolutional neural network to integrate the knowledge of the two more efficiently.
3. We conduct extensive experiments on real world datasets, comparing the proposed DSPose method. Experiments results prove the effectiveness of our model in various aspects. Furthermore, the experiment also proves that modeling the correlation of the human body’s keypoints in physical space can further improve the model’s accuracy.

## 2. Related Work

### 2.1. Human Pose Estimation

Human pose estimation is an important task in computer vision. Briefly, the main goal of the task is to detect the keypoints of the human body in a 2D image and label their locations.

In 2D pose estimation, multi-person pose estimation is the main research task. For this task, there are two main solutions: top–down and bottom–up. Top–down means each person in an image is detected first, and then, the keypoints of each person are identified. It transforms multi-person pose estimation task into multiple single-person pose estimation tasks, while bottom–up means that all the keypoints are detected first and then corresponded to each person separately. The method in this paper belongs to the top–down method.

CNN-based methods have dominated past research. Simple Baselines [4] provides one of the simplest and fastest methods: using ResNet [11] as the backbone and adding an inverse convolutional layer at the end of it to generate a heatmap for keypoints prediction, which ultimately yields considerable results. This demonstrates that methods that work well in other CV tasks also work well in human pose estimation tasks through simple modifications. CPN [5] uses ResNet as the backbone, and the researchers design two networks, GlobalNet and RefineNet, to work together, with the former detecting unobstructed keypoints and the latter detecting occluded keypoints; the idea of processing the datasets, respectively, has been widely borrowed. HRNet [6] is used to divide the portrait into several different dimensional levels by convolution; it predicts the locations of the keypoints by fusing the extracted multi-level information and achieves excellent results. These methods have been recognized as benchmark methods in the field and are widely used in academia as well as industry.

However, the above CNN-based method still has shortcomings in capturing the relationship between long sequence features, and it cannot effectively use the feature information between keypoints. The Transformer-based method can capture the context information better and has better generalization ability for long sequence data.

### 2.2. Pose Estimation with Transformer

Transformer [12], proposed by Vaswani et al. in 2017, has attracted much attention in NLP. Its successful application in NLP has led to passionate research in computer vision. Researchers valued its outstanding ability to understand contextual information, so they modeled the Transformer after the way it is used in NLP, where sliced images are used as context, to apply the Transformer to computer vision [13,14,15,16,17,18]. Among them, Visual Transformer [13] is very representative as a backbone that applies to a wide range of tasks. The method divides the image into small slices with position encoding and puts them into the encoder of the original Transformer together with a class token specialized in learning the information in these slices. Pyramid Vision Transformer [14] adds a multi-level pyramid structure to Vision Transformer, which enables the Transformer to extract information from different scales. Swin Transformer [15] replaces the rigid segmentation of ViT by using moving frames for segmentation, which can capture the relationship between different parts of the image.

In human pose estimation, Transformer-based models have also achieved initial success. Transpose [9] obtained good results by using HRNet as a backbone and inputting the extracted features directly into the Transformer; TokenPose [8] mimicked the design of ViT by adding randomly initialized tokens that represent the keypoints’ features, so the keypoints features are obtained during the Transformer process without subsequent processing; HRFormer [7] embeds the Transformer into the structure of HRNet to replace the original convolution operation and also achieves good results; ViTPose [10] even uses the pure ViT structure directly, with multiple datasets for long-time training, and performs a very high detection accuracy.

Transformer-based human pose estimation methods accurately capture the feature connections between keypoints located at various parts of the image, giving better results with fewer parameters. However, these methods obtain the connections between keypoints only by learning directly from the input image data through multi-layer self-attention, failing to mine the fundamental relations in the human skeleton well enough to provide priori information to assist training. With this in mind, we can view the human skeleton as a graph network and use GNN methods to deal with it.

### 2.3. Graph Neural Network

GNN [19] is a convolutional neural network designed based on graph theory. Unlike CNNs, which focus on pixels that are closely arranged in position, GNNs are mainly used to deal with this situation: elements are related to each other, but the relationships are difficult to describe directly. These elements and relationships are often represented using nodes and edges to form a graph structure, and the GNN performs subsequent operations such as feature aggregation and extraction.

Mimicking the operation of CNN that aggregates the information of surrounding pixels, GCN [20] multiplied the neighbor matrix, which had been transformed by Laplace transform, the feature matrix, and the weight matrix, so that the features between neighboring nodes are aggregated. And for the feature aggregation of GCN, GraphSAGE [21] improved it by proposing two other ways of aggregation: the LSTM aggregation and the pooling aggregation, which aggregated the information of neighboring nodes more scientifically. To make the information of neighboring nodes aggregated according to importance, GAT [22] added the attention mechanism to obtain the features of neighboring nodes selectively.

Because of the success of GNN methods in recommender systems and social networks [23,24,25,26], related research has been gradually conducted in computer vision [27,28,29,30,31,32]. In image annotation, Curve-GCN [28] used GCN for the fine prediction of labeled object contours, which improved the efficiency of image annotation; in image multi-label prediction, Chen et al. [29] uncovered the number of times a combination of labels appeared in an image and combined with GCN to construct a link between multiple labels in an image, which assisted in the prediction of multiple labels; in facial expression recognition; GCANet [32] statistically analyzed the dataset and constructed a graph between AUs, and it used GCN to obtain the relationship between the composition of AUs and the corresponding emotions, which improved the accuracy of expression recognition. To prove that GNNs alone are also effective in processing images, Vision GNN [31] imitated Vision Transformer to perform the segmentation of images and constructed a graph network between these slices to selectively perform feature fusion between the slices, which is more flexible than the traditional method. The above studies show the feasibility and application potential of GNN in computer vision. However, its application in 2D human pose estimation is still rare, and how to use GNN to deal with the possible graph structures in this task is still worth deeply digging into.

## 3. Method

**Figure 1:**
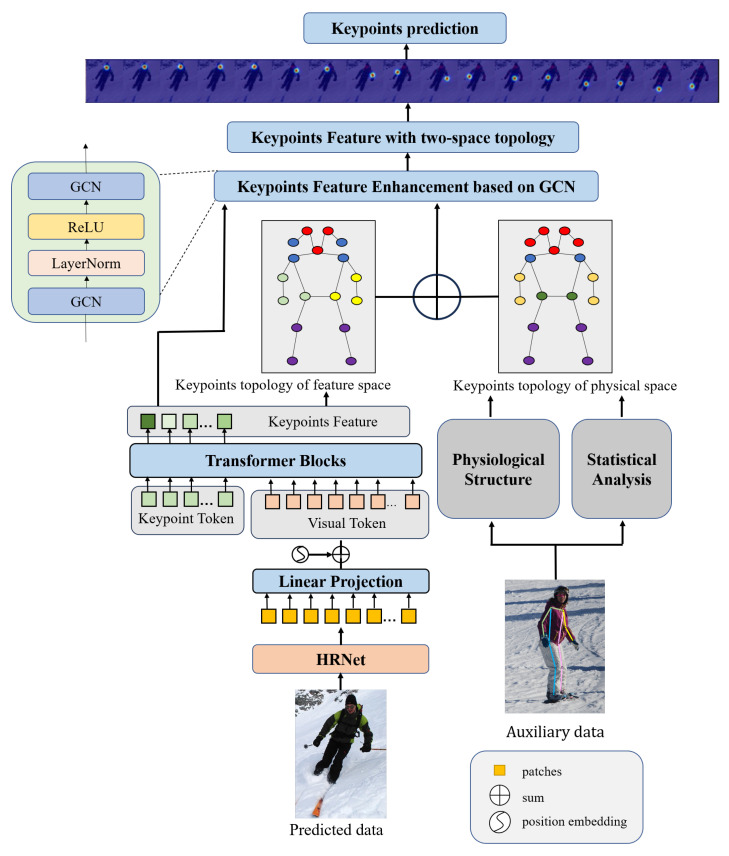
Schematic illustration of DSPose. The method is divided into four stages: 1. Keypoints feature extraction; 2. Keypoints topology modeling in dual space; 3. Keypoints feature enhancement based on GCN; 4. Keypoints prediction. In stage 1, a Transformer-based method is used to extract the keypoints feature, which consists of HRNet, a feature map extraction network, and multi-token Vision Transformer, which is a keypoints feature extraction network. In stage 2, the keypoints topology of physical space is constructed based on the human physiological structure and statistical analysis, which are, respectively, summed up from prior knowledge of what the human body composition and statistical results from datasets. The keypoints topology of feature space is constructed based on the cosine similarities of keypoints with KNN. The topology structures are used to direct the update of keypoints feature. In stage 3, keypoints feature is enhanced by GCN. In stage 4, keypoints feature is transformed into heatmaps for predicting coordinates.

The proposed DSPose framework is shown in Figure 1. The DSPose model consists of three parts. The first part is the keypoint feature representation. Firstly, two types of tokens are constructed based on the input image: Visual Tokens and Keypoint Tokens. Then, the two types of tokens are concatenated as the input of the Transformer to explore the correlation between the keypoints and the image regions and generate the initial keypoint features. The second part is the construction of keypoint topology in dual space. In order to fully represent the keypoint structure relationship, we propose two key point structure construction strategies in physical space and feature space. For the physical space, the keypoints in each image are divided into five clusters based on the human shape structure and prior knowledge. For the feature space, the topology structure is modeled based on the relative position relationship between keypoint features, and the keypoints close to each keypoint feature are selected to establish correlation. Based on the topological structure of keypoints in physical space and feature space, GCN is applied to update the keypoint features continuously. Finally, the keypoint position is represented as a heatmap for training and optimization. The experimental results show that we obtain higher detection accuracy with fewer parameters, and our model design does make a difference.

### 3.1. Keypoints Feature Extraction

The keypoints in the image are located in different regions. Therefore, in order to establish topological relationships between keypoints and multiple regions of the image, the images and keypoints are explicitly embedded as Visual Tokens and Keypoint Tokens to learn constraint relationships and appearance cues from images simultaneously. Then, Visual Tokens and Keypoint Tokens are concatenated as input to the Transformer to learn feature representations of keypoints.

#### 3.1.1. Multi-Type Tokens Construction

**Visual Token.** Inspired by the Vision Transformer, the image is segmented into multiple Visual Tokens. Firstly, HRNet, with the fourth down-sampling removed, was used to extract features of the image and generate feature maps $x\in R^{H\times W\times C}$. Then, split x into $\frac{H}{P_{h}}\times \frac{W}{P_{w}}$ patches of size $P_{h}\times P_{w}$, which is denoted as *p*. Each patch is flattened into a 1D vector with a size of *d*:$f:p\to v\in R^{d}$. Considering the location information of different patches, position embeddings are added to the feature vectors to finally form Visual Tokens: $VT=\{v_{1}+pe_{1},v_{2}+pe_{2},\ldots ,v_{n}+pe_{n}\}$.

**Keypoint Token.** Each keypoint in the image is represented by a learnable d-dimensional vector, called a keypoint token, donated by *k*. Each Keypoint Token feature vector is randomly initialized and denoted as $KT=\{k_{1},k_{2},\ldots ,k_{c}\}$, where *c* represents the number of keypoints in an image.

#### 3.1.2. Transformer Blocks

We concatenate Visual Tokens and Keypoint Tokens as input to the Transformer to learn keypoint features: $U_{0}=\left\{VT,KT\right\}$. The model learns keypoint features through multiple Transformer blocks, and each block consists of three layers: LN, MSA (multi-head self-attention), and LN. Two LN (layer norm) layers are used for token normalization. The self-attention mechanism is calculated as follows:

(1) $$SA\left(U^{l-1}\right)=softmax\left(\frac{U^{l-1}W_{Q}{\left(U^{l-1}W_{K}\right)}^{T}}{\sqrt{d}}\right)\left(U^{l-1}W_{V}\right).$$

In the formula, $W_{Q},W_{K},W_{V}\in R^{d\times d}$ are learnable parameter matrices corresponding to *Q*, *K*, *V*. $U^{l-1}$ is the output of l−1 layers and *d* is the embedding dimension of each token. To enrich the feature representation of Keypoint Tokens from multiple dimensions, the above operation is repeatedly carried out N times through different transformation matrices. The multi-head self-attention mechanism is calculated as follows:

(2) $$MSA\left(U\right)=Concat\left(SA_{1}\left(U\right),SA_{1}\left(U\right),\ldots ,SA_{N}\left(U\right)\right)W_{O},$$

where $W_{O}\in R^{(h\cdot d)\times d}$.

After training with multiple Transformer blocks, keypoint features are extracted by modeling the correlation between Keypoint Tokens and different Visual Tokens, which are specifically expressed as follows:

(3) $$H=\left\{k_{1}^{\prime },k_{2}^{\prime },\ldots ,k_{n}^{\prime }\right\}=Transformer\left(\left\{k_{1},k_{2},\ldots ,k_{n}\right\}\right).$$

### 3.2. Keypoints Topology Modeling in Dual Space

The feature vectors of keypoints are closely related to the image region and limited by the topological structure between keypoints. In order to represent keypoints more effectively, we model the correlation between keypoints from two spaces: physical space and feature space. On the one hand, the topology of keypoints in the physical space is constructed based on the original physiological structure and prior knowledge of the human body. On the other hand, the topological structure is modeled based on the correlation of the feature vectors of keypoints. Finally, GCN was used to comprehensively consider the topological structure of keypoints in the dual space and generate the enhanced keypoint features.

#### 3.2.1. Keypoints Topology Construction of Physical Space

The physiological structure of the human body has certain rules. For example, head is a rigid part of our body covered by less clothing, and the correlation of multiple keypoints such as the nose, eyes, and ears should be very close. Therefore, according to the physiological structure of the human body, we summarize the following principles: (1) the keypoints in the head have a strong correlation; (2) the limbs are pairwise related; (3) parts of the body symmetry are relevant.

In addition to considering the physiological structure of the human body, the corresponding prior knowledge should be summarized through the analysis and statistics of the pose dataset. Wei Tang and Ying Hu [33] summarized the correlation between keypoints of the human body by analyzing the huge amount of data from MPII [34]. Precisely, in this paper, the mutual information between different keypoints is calculated, the correlation degree between keypoints is expressed by numerical magnitude after normalization, and spectral clustering is applied to group each keypoint. The visualization result is shown in the figure, where keypoints of the same color are closely related and have a certain topological structure. It can be seen from the figure that all the keypoints of the human body are divided into six clusters: (1) head top, upper neck, and thorax, (2) left wrist, left elbow and left shoulder, (3) right wrist, right elbow, and right shoulder, (4) left knee and left ankle, (5) right knee and right ankle, (6) left hip, right hip and pelvis. The keypoints in each cluster have a certain topological structure.

**Figure 2:**
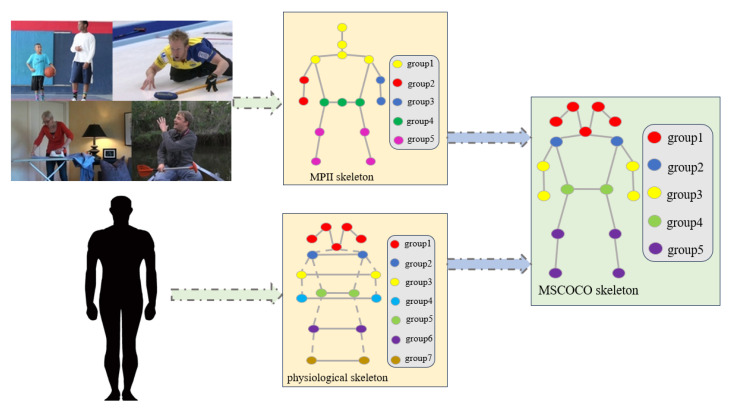
Construction of keypoints topological structure of physical space. The physiological skeleton and MPII skeleton come from the physiological structure and statistics of the MPII dataset, respectively. Then, these two skeletons are combined to construct the MSCOCO skeleton, which contains five groups.

The above prior knowledge is based on the MPII dataset, and the above construction methods can be extended to other datasets. The MSCOCO dataset [35] is another keypoints detection dataset, which contains the nose, left eye, right eye, left ear, right ear, left shoulder, right shoulder, left elbow, right elbow, left hip, right hip, left knee, right knee, left ankle, and right ankle. The former structures should be combined, as shown in Figure 2. According to the results of human physiological structure and statistical analysis, the following modifications are made based on MPII keypoints topology: (1) The five keypoints of the head are grouped. (2) The symmetric keypoints are grouped. (3) The rest remain consistent. In this way, the physical space topology applicable to MSCOCO is obtained. Its correlation is divided into the following five groups: (1) nose, left eye, right eye, left ear and right ear; (2) left shoulder and right shoulder; (3) left elbow, right elbow, left wrist, and right wrist; (4) left hip and right hip, (5) left knee, right knee, left ankle, and right ankle.

The keypoints topological structure of physical space is constructed based on the prior knowledge of the human physiological structure and statistical structure of the dataset. The matrix representing the topological structure consists of solid and weak correlation matrices. The strong correlation matrix highlights the correlation of keypoints in the physiological structure. The strong correlation matrix highlights the correlation of keypoints in the physical space topology. The keypoints in the same group in the figure are strongly correlated and are represented by 1. For example, the relationships within the five groups of keypoints in the figure above, and the remaining relationships between the keypoints are represented by 0. The weak correlation matrix guarantees that there is information exchange between all keypoints and is an all-1 matrix of c×c. Its formula is as follows:

(4) $$A_{p}=a\cdot A_{s}+b\cdot A_{w},$$

where $A_{p}$ is the matrix used to represent the physical space topological relationship of keypoints, $A_{s}$ is the strong correlation matrix, $A_{w}$ is the weak correlation matrix, *a*, *b* are the weights, and they satisfy a∈(0.7,1), b∈(0,0.3), a+b=1. The above matrix represents the true association of the keypoints.

#### 3.2.2. Keypoints Topology Construction of Feature Space

**Figure 3:**
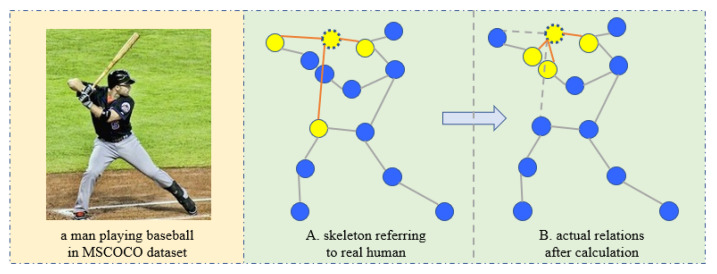
Keypoints relationships in images may differ from those in the real world. (**A**). Based on real-world knowledge, the left shoulder is most closely associated with the left elbow, left hip, and neck. (**B**). After calculating the keypoint similarity, those most closely associated with the left shoulder are the left wrist, the right wrist, and the neck.

Keypoints relations sometimes do not obey the topological structure of physical space, as shown in Figure 3. In addition to considering the topological structure of keypoints in the physical space, we also propose a method to construct the topological structure of keypoints in the feature space. According to the Keypoint Token feature vectors output by Transformer, the cosine similarity between each pair is calculated, and a similarity matrix S is formed to represent the correlation between keypoints. The specific calculation formula is as follows:

(5) $$S_{mn}=\frac{x_{m}x_{n}}{∥x_{m}∥∥x_{n}∥}\left(m,n=1,2,\ldots ,c\right),$$

where $S_{mn}$ represents the element of the $m^{th}$ row and $n^{th}$ column in the similarity matrix, $x_{m}$ and $x_{n}$ represent the feature vectors of the m and n keypoints, respectively, and c represents the number of keypoints.

In order to distinguish the strong and weak associations between keypoints, the t largest similarities (including the similarities calculated with itself) are kept in each row of the similarity matrix, and the remaining positions are set to 0.

(6) $$a_{in}=\left\{\begin{array}{cc} s_{mn} & i=m,s_{mn}\in Topt\left(s_{in}\right)\\ 0 & i=m,s_{mn}\notin Topt\left(s_{in}\right) \end{array}\right..$$

(7) $$A_{f}=\left(\begin{array}{ccc} a_{11} & \ldots & a_{1n}\\ \vdots & \ddots & \vdots \\ a_{i1} & \cdots & a_{in} \end{array}\right),$$

where $a_{in}$ represents a single element in the similarity matrix. $A_{f}$ represents the similarity matrix of the topological structure of keypoints in the feature space.

### 3.3. Keypoints Feature Enhancement Based on GCN

To comprehensively characterize the topological relationships among keypoints, we fused the topological structure of keypoints in the physical space and in the feature space to generate the final keypoints topological structure. The fusion process represented by the adjacency matrix is as follows:

(8) $$A_{m}=p\cdot A_{p}+A_{f},$$

where $A_{m}$ represents the final keypoints topological structure and *p* represents a learnable weight matrix, which adaptively adjusts the proportion of the two topological structures in the fusion process.

In order to make the extracted keypoints feature more accurate, GCN and the keypoints topological structure constructed above are used to update the keypoints feature. The keypoints feature matrix $H^{\left(0\right)}=H\in R^{c\times c}$ is the input of GCN, and the keypoints topological structure $A_{m}\in R^{c\times c}$ is the adjacency matrix that guides the keypoints feature updating in GCN. The update process goes through multiple layers of GCN. In layer *l*, feature $H^{\left(l\right)}$ that needs to be updated is first normalized by layernorm, and then, it is fused in GCN. Finally, the nonlinear activation function is activated to obtain the updated feature $H^{\left(l+1\right)}$. Its formula is expressed as follows:

(9) $$H^{\left(l+1\right)}=\sigma \left(A_{m}\left(LN\left(H^{\left(l\right)}\right)\right)W^{\left(l\right)}\right),$$

where σ stands for nonlinear activation function and LN stands for layernorm. After *N* layers GCN, the updated keypoints feature matrix $H^{f}$ is obtained.

### 3.4. Keypoints Prediction

Based on the keypoints feature, the heatmaps of size H*W are generated for the keypoints location prediction. Firstly, the one-dimensional keypoints feature is transformed into a two-dimensional matrix of H*W by ascending dimension to represent the heatmap, in which the data at different positions in the thermal map represent the probability of the keypoint occurrence there: $f:V^{\left(c\times (h\cdot w)\right)}\to M^{\left(c\times h\times w\right)}$, where *V* represents the keypoints feature, *M* represents the heatmaps, *c* represents the number of keypoints, and *h* and *w* represent the height and width of the heatmap.

In the training and optimization stage of DSPose, MSE loss is used as a loss function. This is a common loss function, and its principle formula is as follows:

(10) $$MSE=\frac{1}{n}\sum_{i=1}^{n}{\left(Y_{i}-{\hat{Y}}_{i}\right)}^{2},$$

where *n* represents the number of values in the predicted heatmap, $Y_{i}$ represents the value of the corresponding position in the predicted heatmap, and ${\hat{Y}}_{i}$ represents the value of the corresponding position in the ground-truth heatmap.

## 4. Experiments

This section describes the training setup of the model on the COCO keypoints detection dataset and MPII human pose dataset, the comparison of the results with the SOTA methods on COCO validation, test-dev sets, MPII validation set, the ablation study, and the model visualization.

**Dataset.** The **COCO keypoints detection dataset** contains over 200,000 images and 250,000 portraits. Each of these portraits is labeled with 17 keypoints, which are the nose, left eye, right eye, left ear, right ear, left shoulder, right shoulder, left elbow, right elbow, left hip, right hip, left knee, right knee, left ankle, and right ankle. The COCO keypoints detection dataset is divided into a training set, a validation set, and a test set, which contain 118,000, 5000, and 20,000 images, respectively.

The **MPII Human Pose Dataset** contains 25,000 tagged images of more than 40,000 portraits. These images are taken from Internet videos. Each portrait contains 16 keypoints, which are the right ankle, right knee, right hip, left hip, left knee, left ankle, pelvis, thorax, upper neck, head top, right wrist, right elbow, right shoulder, left shoulder, left elbow and left wrist.. There are 22,000 and 3000 images for training and testing.

**Evaluation metrics.** For COCO keypoints detection, we used average precision (AP) as the evaluation metric for model effectiveness. AP is calculated based on OKS with the formula: OKS = $\frac{\sum_{i}{\exp(-\overset{⌢}{\;d}}_{i}^{2}/2s^{2}k_{i}^{2})\sigma \left(v_{i}>0\right)}{\sum_{i}\sigma \left(v_{i}>0\right)}$, where ${\hat{d_{i}}}^{2}$ denotes the Euclidean distance between the predicted coordinates and the true coordinates of a keypoint, *v_i_* represents whether the key point is occluded or not, *s* represents the object scale, and *k_i_* represents the constraints of the keypoint. OKS indicates how close the predicted key points are to the ground truth. AP is calculated by OKS: $AP=\frac{\sum_{p}\delta (oks_{p}>T)}{\sum_{p}1}$, where *p* represents the person *p* in the data set and *T* represents the threshold. When *T* = 0.5, it is *AP*^50^; when *T* = 0.75, it is *AP*^75^. For MPII keypoints detection, we use PCK@0.5 as the evaluation metric, which means the percentage of detections that fall within a normalized distance of the ground truth, and the threshold is 0.5.

**Implementation details.** The model parameters used in all our experiments are shown in Table 1. The model follows a top–down training approach, where people in the image are first detected and formed into a uniformly sized detection frame. The size of the detection frame is 256 × 192, and the human detection method is the same as the method used in SimpleBaseLine. The optimization algorithm for the model in the experiment is Adam’s algorithm. During the training process, the batch size of each GPU is set to 32, and the initial learning rate is 1 × 10${}^{-3}$, which decreases to 1 × 10${}^{-4}$ at the 200th epoch, and 1 × 10${}^{-5}$ at the 260th epoch, for a total of 300 epochs. For model evaluation after training, DARK [36] is used as the heatmap decoding method. The experiments were performed according to their original settings for the SOTA methods.

In the following article, we use some symbols to denote the hyperparameters. N denotes the number of hidden layers in the GCN, and t denotes the largest value in each row of the adjacency matrix of the feature space topology relations. Usually, we set *N* = 2, *t* = 4.

**Comparison Method**

**SimpleBaseline.** The runner-up solution of the COCO human pose estimation competition was presented in 2018. It is a simple and efficient method using the ResNet series as the backbone in combination with the inverse convolution for the output prediction. SimpleBaseline with the backbone of ResNet-50, ResNet-101, and ResNet-152 is used for comparison in this experiment.

**HRNet.** A backbone proposed by Microsoft Research Asia for multi-domain tasks. The method down-samples the feature map layer by layer, allowing different scale features to interact, giving the model a powerful feature extraction capability. In this experiment, two models HRNet-W32 and HRNet-W48 are used for comparison.

**TokenPose.** A Transformer-based pose estimation method, one of the first to use Transformer in human pose estimation. TokenPose-B is chosen for comparison to harmonize its backbone with the method in this paper.

**G-RMI [37].** The cornerstone of human pose estimation, proposed in 2017, identifies the steps of the top–down approach: figure detection in images using Faster-RCNN first, followed by keypoint detection using ResNet.

**Integral Pose Regression [38].** A human pose estimation technique was proposed in 2018. The technique combines the heatmap and regression methods using a simple integration operation. It improves the detection accuracy of the thermogram regression method in low-resolution images.

**CPN.** The champion method for the COCO keypoint detection challenge was presented in 2017. The method expands on ResNet with two interconnected networks: GlobalNet and RefineNet. The former detects unobscured keypoints, and the latter detects occluded keypoints. This classification and detection idea brings good results.

**RMPE [39].** A top–down pose estimation method proposed in 2017. The method mainly focuses on the human detection phase. It uses a symmetric spatial transformation network to improve the quality of the human detection frame while applying parametric pose non-maximization suppression to solve the redundancy detection problem.

**PRTR [40].** A human pose estimation method based on Transformer released in 2021. PRTR is a regression method that uses a cascade Transformer to achieve SOTA of this kind of method.

**UniFormer [41].** A proposed backbone network combining CNN and Transformer in 2022. The network borrows from CNN’s layered design but contains three small layers within each layer in the style of Transformer. This approach goes one step further in becoming the backbone of most downstream tasks.

**DistilPose [42].** Human pose estimation method proposed in 2023. The method focuses on keypoints encoding and decoding. It uses a token extraction encoder combined with the heatmap and regression method and an analog heatmap to achieve efficient keypoints encoding and decoding.

**DPIT [43].** Transformer-based approach proposed in 2022. This method combines the top–down and bottom–up methods to extract features as input to the Transformer, which has a certain improvement in keypoint detection compared with previous methods.

**TFPose [44].** Human pose estimation method based on Transformer proposed in 2021. This method is one of the first to use Transformer for key point coordinate regression, which greatly improves over previous regression methods.

**SimCC [45].** A new method to characterize keypoints in the human body was published in 2022. This method improves the representation of keypoints by using two one-dimensional vectors instead of thermal maps to represent the coordinates of keypoints, which is helpful for both CNN and Transformer methods in human body pose estimation.

### 4.1. COCO Keypoints Detection

**Comparison of test results.** As shown in Table 2, DSPose achieves better results than the previous SOTA methods with fewer parameters. Compared to the SimpleBaseLine series, DSPose still achieves 3.1% higher AP and 2.4% higher AR than SimpleBaseline-Res152 with very few parameters. Compared with HRNet-W32, which also has 32 input channels, DSPose improves on all indicators except for a slight shortfall on $AP^{50}$. The most improvement is 1.0% on AR. Compared with TokenPose-Base, except for $AP^{50}$, DSPose achieves certain improvement on all other metrics, and the improvement is 0.4%, 0.6%, 0.2%, 0.2%, 0.6%, 0.2%, and 0.2%, respectively. Our analysis suggests that the $AP^{50}$ of DSPose is reduced compared to HRNet due to the further suppression of forced fitting of images with poor quality, which results in the abandonment of images that may meet the criteria under looser detection criteria. However, because of this suppression, DSPose can locate the keypoints more accurately in acceptable-quality images. This conclusion was also verified on the test set. We then tested the model on the coco2017 test-dev dataset. The results are shown in Table 3, and it can be seen that with the same input data size and number of parameters, the model has improved in all accuracy metrics except for $AP^{50}$. The model is even close to HRNet-W48, which has a much larger number of parameters, with HRNet-W32 as the backbone. The above experimental results further demonstrate the effectiveness of DSPose in suppressing overfitting and improving the overall detection accuracy.

### 4.2. MPII Keypoints Detection

**Comparison of test results.** As shown in Table 4. DSPose achieves very competitive results in MPII keypoints detection tasks with minimal parameters. Out of the eight indicators, DSPose achieved four of the best and four of the second best, proving that it has excellent results for different data sets.

### 4.3. Ablation Study

**Topology of physical space and feature space.** In our model, constructing the topology of the physical space and the feature space is one of the biggest innovations. To refine their roles in improving the model’s accuracy, we performed relevant ablation experiments. We sequentially trained the network with only the topology of feature space added and then both together. And we tested their detection accuracy on the coco2017 val dataset. The results are shown in Table 5. It can be seen that using topological relations in feature space plus GCN has a significant improvement in AP and AR over the commonly used MLP alone for output, while adding topological relations in physical space again leads to a small increase in detection accuracy. The results show that our method better utilizes the priori and feature information of the human skeleton and is more suitable for this task.

We also conducted the same experiment on the MPII dataset. The MPII dataset has the following characteristics: (1) relatively small amount of data; (2) excerpted from video; keypoints of human bodies in different images may have similar relations; (3) the people in the videos are mostly centered, and the shooting is more clear. In this case, as shown in Table 6, the effect of using only the feature space adjacency matrix is less than that of MLP, while the effect of using the physical space and the feature space adjacency matrix is greater than that of the former. We analyzed the correlation with the data set for the opposite results of the ablation experiments on COCO and MPII. For COCO, a dataset with large quantities and uneven quality, the feature space adjacency matrix plays a major role in fitting various cases. For MPII, with a relatively small amount of data and clear character characteristics, the physical space adjacency matrix can already fit the keypoints relationships well and guide the feature extraction of keypoints.

**Figure 4:**
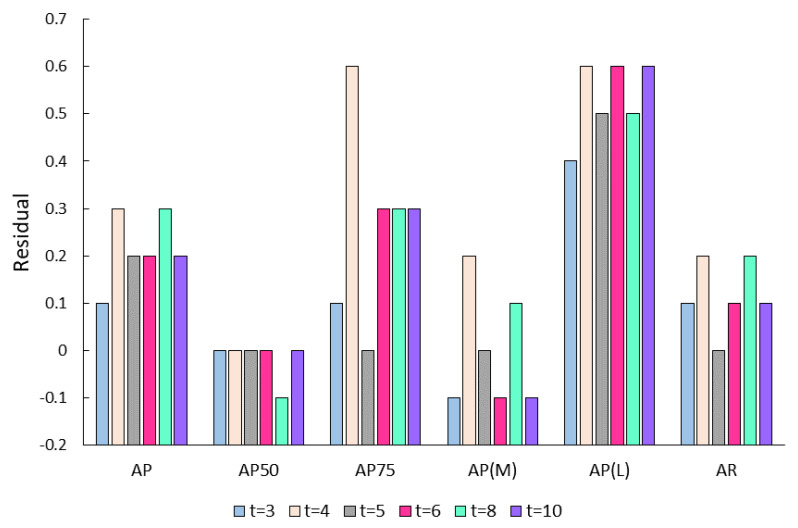
Residual of DSPose when t takes different values.

**Topological composition of the feature space.** When constructing the topological relationships of the feature space in our model, we use the feature vectors to compute the similarity matrix and choose a few keypoints with the highest similarity to each keypoint to form the edges of the adjacency matrix. The number of these keypoints with the highest similarity needs to be carefully considered as an important hyperparameter. The experiment’s result shows the model’s accuracy when different *t* values are taken, as shown in Table 7. It can be seen that the accuracy is different when different *t* values are taken. In order to compare the accuracy when *t* is different and select the optimal *t*, we plot the following line graph Figure 4, where the vertical coordinate represents the degree of improvement of each evaluation index over TokenPose-B. The line graph shows that the overall accuracy improvement is greatest at *t* = 4, while smaller or larger values do not achieve optimal accuracy.

**Quantity of GCN layers.** The quantity of GCN layers also affects the accuracy. In CNNs incorporating residual connectivity, the more CNN layers there are, the finer the information learned by the model will be and the higher the model accuracy. However, this is not necessarily the case in GCN. It has been shown that the optimal quantity of GCN layers is 2 or 3. To verify how many GCN layers are appropriate in this model, we set the quantity of GCN layers to 1, 2, and 3 for the experiments, respectively. Since the model parameters increase substantially as the quantity of GCN layers increases, if there is no significant improvement in accuracy with the increasing quantity of layers, it can be determined that there is no need to increase the number of layers. The experimental results are shown in Table 8. It can be seen that from *N* = 1 to *N* = 2, AP and AR both have significant improvement, but from *N* = 2 to *N* = 3, there is no improvement, so the best number of GCN layers is 2 according to the experiment.

### 4.4. Visualization

**Figure 5:**
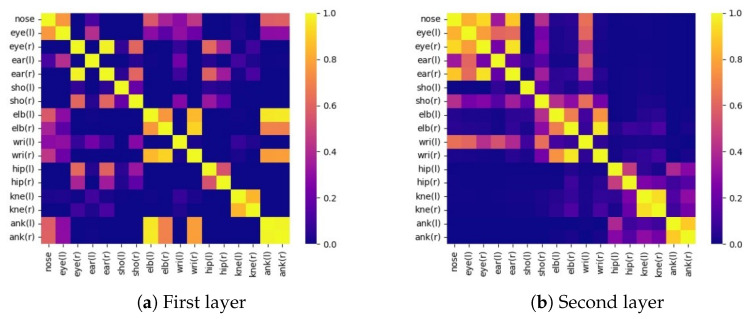
Cosine similarity matrices of keypoints after GCN. (**a**) After the first layer, some physical spatial associations that fit the analysis have been found. (**b**) After the second layer, most of the associations obtained by the analysis turned out to be correct.

**The learning process of graph convolutional networks.** In order to visualize the learning process of graph convolutional networks, we have calculated the cosine similarity of the feature vectors output from the two layers of GCN and presented them in the form of a heatmap, as shown in Figure 5. It can be seen that after the first layer of GCN, the keypoint features are already different from each other to a certain extent, showing some topological relationships in line with the physical space constructed in the text: for example, hips, knees, ankles have been paired up, and elbows have been associated with wrists; at the same time, some of the features are not yet fully differentiated, resulting in some undesirable correlations. After the second layer of GCN, the correlation and differentiation of keypoint features are more obvious. In the heatmap, the five points of the head are highly correlated, the symmetry points of the rest of the positions are similar, and there are some additional correlations for the upper body. This result is consistent with our construction of physical topological relationships for keypoints and demonstrates the adaptation of feature space topological relationships to the realities of the image.

**Figure 6:**
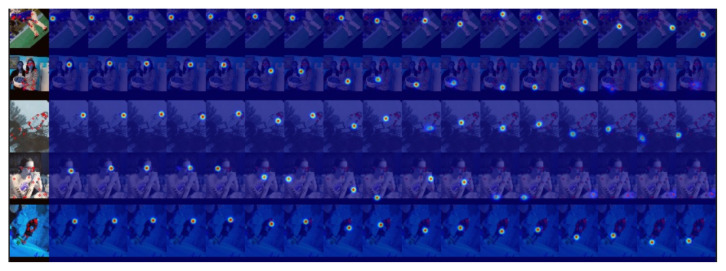
Visualization of the keypoints in the form of a heatmap. In the case of blurred images, ghosts, and the overlapping of two human bodies, DSPose can still accurately predict the coordinates of keypoints.

**Figure 7:**
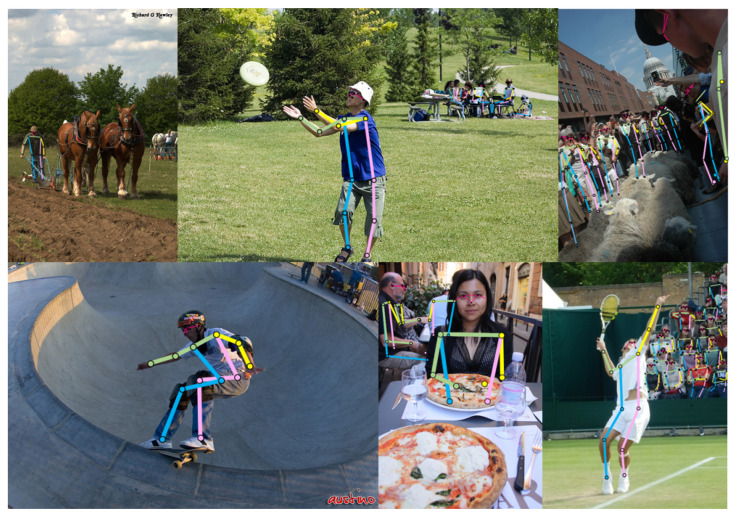
Human pose estimation results of DSPose. DSPose has achieved excellent estimation results when dealing with different sizes of the human body, different postures of the human body, and different occlusion conditions, which shows the universality of this method in multi-source data.

**Visualization of detection results.** We visualized the predicted heatmaps as well as the keypoints obtained from the final decoding, as shown in Figure 6 and Figure 7. The training data were processed by rotating, scaling, and blurring based on the COCO training set. From the figure, it can be seen that in the face of the images after these processing steps, our method can still find the location of the keypoints and generate the heatmaps accurately, which shows that DSPose has high accuracy and strong robustness. By analyzing the skeleton maps of keypoints generated after decoding, it can be seen that our method can adapt to different lighting conditions, pose, and size. At the same time, it also has an outstanding adaptability to the situation where the keypoints are blocked, and it can detect the location of keypoints more accurately.

## 5. Conclusions

In this paper, we propose a dual-space-driven human pose estimation method called DSPose, which can effectively combine the correlation of keypoints in the physical space and the correlation of the feature level to obtain accurate pose estimation results. Specifically, the features of keypoints are extracted by a Transformer-based feature extractor. Then, the association of keypoints in physical space is obtained based on human physiological structure and statistical analysis method, and the association of keypoints in feature space is obtained by calculating similarity based on keypoints features. Finally, the correlation of the physical space and the correlation of the feature space are effectively fused to guide the keypoints features update in GCN, and more accurate keypoints features and coordinates are obtained. Our model achieves superior performance with fewer parameters compared to the state-of-the-art methods. This work will provide a new perspective on the problem of accurate localization in human pose estimation. The experiment also proves that the correlation of physical space is very important for accurately positioning human keypoints.

In the course of the experiments, we also found that the portraits in the datasets were incomplete. This situation will cause some keypoints to be vacant, affecting the effect of GCN keypoint update. At the same time, the portraits in the datasets can often be divided into multiple categories according to their specific scenes, and the keypoints association rules of the portraits in different categories will be slightly different. However, in our model, we build a physical space topological relationship for all portraits, which is insufficient in the case of dealing with multiple types of data. In the future, we will add different categories of datasets to improve the construction of physical space adjacency matrices. Specifically, we will classify the characters in the datasets according to their completeness and action categories, and we will construct different physical space adjacency matrices for each category to make the obtained physical space topological relations more targeted so as to further improve the accuracy of the model.

## Figures and Tables

**Table 1:** Architecture configurations. The model parameters are computed under an image with 256 × 192 input resolution.

Model	CNN Backbone	Transformer Layers	Embedding Size	Heads	Patch Size	Params
DSPose-B	HRNet-W32-stage3	12	192	8	4 × 3	13.5 M
DSPose-L	HRNet-W48-stage3	6	192	8	4 × 3	20.8 M

**Table 2:** Comparisons on the COCO validation set provided with the same detected human boxes. Our model achieves competitive results compared to the state-of-the-art models. Bold indicates the optimal value of the indicator, underline indicates the sub-optimal value of the indicator.

Method	Params	AP	$AP^{50}$	$AP^{75}$	$AP^{M}$	$AP^{L}$	AR
SimpleBaseline-Res50	34.0 M	70.4	88.6	78.3	67.1	77.2	76.3
SimpleBaseline-Res101	53.0 M	71.4	89.3	79.3	68.1	78.1	77.1
SimpleBaseline-Res152	68.6 M	72.0	89.3	79.8	68.7	78.9	77.8
PRTR	57.2 M	73.3	89.2	79.9	69.0	80.9	**80.2**
UniFormer-S	25.2 M	74.0	90.3	**82.2**	66.8	76.7	79.5
DistilPose-L	21.3 M	74.4	89.9	81.3	71.0	81.8	-
HRNet-W32	28.5 M	74.4	**90.5**	81.9	70.8	81.0	79.8
TokenPose-B	**13.5 M**	74.7	89.8	81.4	71.3	81.4	80.0
DSPose-B	**13.5 M**	**75.1**	89.8	82.0	**71.5**	**82.0**	**80.2**

**Table 3:** Comparisons on the COCO test-dev set with state-of-the-art models. Bold indicates the optimal value of the indicator, underline indicates the sub-optimal value of the indicator.

Method	Input Size	Params	AP	$AP^{50}$	$AP^{75}$	$AP^{M}$	$AP^{L}$	AR
G-RMI	353 × 257	42.6 M	64.9	85.5	71.3	62.3	70.0	69.7
Integral Pose Regression	256 × 256	45.0 M	67.8	88.2	74.8	63.9	74	-
CPN	384 × 288	-	72.1	91.4	80	68.7	77.2	78.5
TFPose	384 × 288	20.4 M	72.2	90.9	80.1	69.1	78.8	-
RMPE	320 × 256	28.1 M	72.3	89.2	79.1	68.0	78.6	-
DPIT-B	256 × 192	20.8 M	73.6	91.4	81.2	70.4	79.5	78.9
DistilPose-L	256 × 192	21.3 M	73.7	91.6	81.1	70.2	79.6	-
SimpleBaseline-Res152	384 × 288	68.6 M	73.7	91.9	81.1	70.3	80.0	79.0
HRNet-W48	256 × 192	63.6 M	74.2	**92.4**	**82.4**	70.9	79.7	79.5
TokenPose-B	256 × 192	**13.5 M**	74.0	91.9	81.5	70.6	79.8	79.1
DSPose-B	256 × 192	**13.5 M**	**74.5**	91.9	82.2	**71.1**	**80.4**	**79.6**

**Table 4:** Comparisons on the MPII validation set with SOTA and recent methods. Bold indicates the optimal value of the indicator, underline indicates the sub-optimal value of the indicator.

Method	Params	Hea	Sho	Elb	Wri	Hip	Kne	Ank	Mean
SimpleBaseline-Res50	34.0 M	96.4	95.3	89.0	83.2	88.4	84.0	79.6	88.5
SimpleBaseline-Res101	53.0 M	96.9	95.9	89.5	84.4	88.4	84.5	80.7	89.1
PRTR	57.2 M	**97.3**	**96.0**	90.6	84.5	**89.7**	85.5	79.0	89.5
SimpleBaseline-Res152	68.6 M	97.0	95.9	90.0	85.0	89.2	85.3	81.3	89.5
SimCC	-	97.2	**96.0**	90.4	85.6	89.5	85.8	81.8	90.0
HRNet-W32	28.5 M	96.9	**96.0**	90.6	85.8	88.7	**86.6**	82.6	90.1
TokenPose-L/D6	21.4 M	97.1	95.9	**91.0**	85.8	89.5	86.1	**82.7**	90.2
DSPose-L	**20.9 M**	**97.3**	**96.0**	90.9	**85.9**	89.5	86.3	82.6	**90.3**

**Table 5:** Evaluation of the two topological structures on COCO validation set.

Method	AP	AR
MLP	74.7	80.0
Feature-A	75.0	80.1
Feature-A + Physical-A	**75.1**	**80.2**

**Table 6:** Evaluation of the two topological structures on MPII validation set.

Method	Hea	Sho	Elb	Wri	Hip	Kne	Ank	Mean
MLP	97.1	95.9	**91.0**	85.8	**89.5**	86.1	**82.7**	90.2
Feature-A	97.1	95.9	90.7	85.6	89.2	86.0	82.5	90.1
Feature-A + Physical-A	**97.3**	**96.0**	90.9	**85.9**	**89.5**	**86.3**	82.6	**90.3**

**Table 7:** Evaluation of our model on COCO validation set with different parameters.

*t*	AP	$AP^{50}$	$AP^{75}$	$AP^{M}$	$AP^{L}$	AR
3	74.8	89.8	81.5	71.2	81.8	79.9
4	**75.0**	**89.8**	**82.0**	**71.5**	**82.0**	**80.2**
5	74.9	89.8	81.4	71.3	81.9	80.0
6	74.9	89.8	81.7	71.2	82.0	80.1
8	75.0	89.7	81.7	71.4	81.9	80.2
10	74.9	89.8	81.7	71.2	82.0	80.1

**Table 8:** Evaluation of our model on COCO validation set with different *N* values.

*N*	Param	AP	AR
1	12.9 M	74.9	80.0
2	13.5 M	75.1	80.2
3	15.8 M	75.0	80.2

## Data Availability

The data used to support the findings of this study are available from the corresponding author upon request.
